# Insulin Resistance Distribution and Cut-Off Value in Koreans from the 2008-2010 Korean National Health and Nutrition Examination Survey

**DOI:** 10.1371/journal.pone.0154593

**Published:** 2016-04-29

**Authors:** Kyung-Jin Yun, Kyungdo Han, Mee Kyoung Kim, Yong-Moon Park, Ki-Hyun Baek, Ki-Ho Song, Hyuk-Sang Kwon

**Affiliations:** 1 Division of Endocrinology and Metabolism, Department of Internal Medicine, Yeouido St. Mary’s Hospital, Seoul, Korea; 2 Department of Medical Statistics, College of Medicine, The Catholic University of Korea, Seoul, Korea; 3 Department of Epidemiology Branch, National Institute of Environmental Health Sciences, National Institutes of Health, Research Triangle Park, North Carolina, United States of America; INRCA, ITALY

## Abstract

**Background:**

We sought to identify the distribution and cut-off value of the ‘homeostasis model assessment of insulin resistance’ (HOMA-IR) according to gender and menopausal status for metabolic syndrome in Koreans.

**Methods:**

Data were from the Korean National Health and Nutrition Examination Survey in 2008–2010. The subjects included adults aged 20 years or older. We excluded participants who had diabetes or fasting serum glucose ≥ 7 mmol/L. Finally, 11,121 subjects (4,911 men, 3,597 premenopausal women, 2,613 postmenopausal women) were enrolled. The modified Adult Treatment Panel III criteria were used to define metabolic syndrome.

**Results:**

The mean HOMA-IR was 2.11 (2.07–2.15) for men, 2.0 (1.97–2.04) for premenopausal women, and 2.14 (2.2–2.19) for postmenopausal women. The first cut-off values in men, premenopausal women, and postmenopausal women were 2.23 (sensitivity 70.6%, specificity 66.9%), 2.39 (sensitivity 72.3%, specificity 76.4%), and 2.48 (sensitivity 51.9%, specificity 80.2%), respectively. Based on the first HOMA-IR cut-off value, the prevalence of metabolic syndrome was 22.9% in men, 13.7% in premenopausal women, and 51.6% in postmenopausal women. The second cut-off value was around 3.2 in all three groups. Based on the second HOMA-IR cut-off value, the prevalence of metabolic syndrome was 50.8% in men, 42.5% in premenopausal women, and 71.6% in postmenopausal women.

**Conclusion:**

In conclusion, the first cut-off values for HOMA-IR were 2.2–2.5 and the second cut-off value was 3.2 in Korea. The distribution of HOMA-IR showed differences according to gender and menopausal status. When we apply HOMA-IR, we should consider gender, menopausal status, and the prevalence of metabolic syndrome.

## Introduction

Insulin resistance (IR) is closely associated with cardiovascular risk factors, including diabetes mellitus, dyslipidemia, and hypertension [1]. Therefore, identifying and quantifying insulin resistance is important, and it can instill awareness in patients. The gold standard method for insulin sensitivity and resistance is the hyperinsulinemic-euglycemic glucose clamp technique [2]. However, this is not readily applied in large-scale investigations because of its complex process. Alternative and simpler methods of measuring insulin resistance include the fasting insulin level, the ‘homeostasis model assessment of insulin resistance’ (HOMA-IR), and the quantitative insulin sensitivity check index (QUICKI) [3, 4].

HOMA-IR is a widely used index and a useful assessment of IR [5]. However, it is still unclear what cut-off value of HOMA-IR should define IR. Several studies for defining cut-off values of HOMA-IR have been published [6–10]. The values were not consistent, and showed gender and racial differences. In 7,057 healthy Korean people (4,472 men, 2,585 women), a high HOMA-IR (≥ 2.56) and fasting insulin (≥ 9.98 μIU/mL) were significantly associated with metabolic syndrome after adjusting for age, gender, and body mass index (*P* < 0.001) [11]. In another study, the cut-off values of HOMA-IR for metabolic syndrome in Korean non-diabetic adults were 2.34 (sensitivity 62.8%, specificity 65.7%) [12]. However, this study was based on single center data and did not distinguish patients by gender.

The purpose of the present study using data representing the Korean population was to identify the distribution of HOMA-IR and to define the cut-off values according to gender and menopausal status for metabolic syndrome.

## Material and Methods

### Subjects

The data for our study were from the Korean National Health and Nutrition Examination Survey (KNHANES) in 2008–2010. The KNHANES is conducted annually by the Korean Ministry of Health and Welfare to monitor the general health and nutritional status of the South Korean population. This survey is composed of a health interview survey, a health examination survey, and a nutrition survey by trained investigators. Data are collected using a rolling sampling design that involves complex, stratified, and multistage probability samples. All participants signed an informed consent form and this survey was approved by the institutional review board of the Korea Centers for Disease Control. Additional details about the survey have been provided elsewhere [13]. The subjects for the present study included adults aged 20 years or older. We excluded participants who had diabetes or fasting serum glucose ≥ 7 mmol/L. Participants with missing data, such as fasting glucose, fasting insulin, and lipid profile were also excluded. Finally, 11,121 subjects (4,911 men, 6,210 women) were included in our analysis.

### Measurement and classification of variables

Body weight and height were measured with the subject wearing light clothing and body mass index (BMI) was calculated using the formula: BMI = weight (kg) / height (m^2^). Waist circumference (WC) was measured at the level midway between the costal margin and the iliac crest at the end of a normal expiration. The subjects were required to rest for at least 5 min before measuring blood pressure using a mercury sphygmomanometer (Baumanometer; Baum, Copiague, NY, USA) in the sitting position. Blood pressure (BP) was measured three times and the mean value of the second and third measurements was used for the analysis.

Blood sampling was performed after at least an 8-h fast. Fasting blood glucose and cholesterol were measured using a Hitachi automatic analyzer 7600 (Hitachi, Tokyo, Japan). Serum insulin was estimated using a radioimmunoassay method with 1470 WIZARD gamma counter (Perkin-Elmer, Turku, Finland). Insulin resistance was measured using HOMA-IR, calculated as follows: HOMA-IR = fasting insulin (μU/mL) × fasting glucose (mg/dL) / 22.5. HOMA-IR was divided into 10 deciles.

Self-reported questionnaires were used to determine menopausal status, smoking status, alcohol consumption, and exercise habit. Premenopausal women were defined as women without a history of reproductive surgery and having > 1 menstruation during the past 12 months. Postmenopausal women were defined as women having no menstruation during the past 12 months. Current smoking was defined as subjects who were currently smoking and had smoked more than 100 cigarettes in their lifetime. The amount of alcohol consumed (g/day) was determined by the amount and type of alcohol for a month. Heavy drinking was defined as the subject drinking more than 30 g/day. Regular exercise was defined as moderate exercise for longer than 30 min at a time at least five times per week, or intense exercise for longer than 20 min at a time at least three times per week.

### Definitions of metabolic syndrome

Metabolic syndrome, reflecting cardiovascular risk factors, has been defined using several criteria. We used the modified Adult Treatment Panel III (ATP III) criteria to define metabolic syndrome. Specifically, metabolic syndrome was defined as the presence of three or more of the following;1) abdominal obesity (WC ≥ 90 cm in men and ≥ 80 cm in women, by World Health Organization-Asian Pacific region criteria [14]), 2) triglycerides ≥ 1.69 mmol/L or on drug treatment for elevated triglycerides, 3) HDL-cholesterol < 1.03 mmol/L (men) or < 1.29 mmol/L (women) or on drug treatment for reduced HDL-cholesterol, 4) BP ≥ 130/85 mmHg or on antihypertensive drug treatment, and 5) fasting plasma glucose ≥ 5.6 mmol/L or on drug treatment for elevated glucose [15].

### Statistical analysis

The data are presented as means ± standard errors (SE) for continuous variables and as proportions (SE) for categorical variables. Analysis of variance (ANOVA) or the chi-square test was used to compare the clinical characteristics between the men, premenopausal women, and postmenopausal women in Table 1. If necessary, logarithmic transformations were performed for variables with skewed distributions. A receiver operating characteristic (ROC) curve was calculated to evaluate HOMA-IR cut-off value for metabolic syndrome. The Youden index, calculated as (sensitivity + specificity—1) was estimated to determine optimal cut-off values. After those subjects with a HOMA-IR lower than the first HOMA-IR cut-off value had been excluded, the data were recalculated to obtain the second cut-off value. The odds ratios (OR) and 95% confidence intervals (CI) were calculated to identify the risk of metabolic syndrome components at the HOMA-IR cut-off level. All values were adjusted by age, BMI and lifestyle factors (current smoking, heavy drinker, regular exercise). All statistical analyses were performed with the SAS software (ver. 9.3; SAS Institute; Cary, NC, USA). A *P*-value < 0.05 was considered to indicate statistical significance.

**Table 1:** Baseline clinical characteristics of the study population.

	Men	Premenopausal women	Postmenopausal women	P-value
N	4,911	3,597	2,613	
Age (years)	42.5±0.3	35.2±0.2	61.7±0.3	<.001
BMI (kg/m^2^)	24.0±0.1	22.5±0.1	24.1±0.1	<.001
WC (cm)	83.9±0.5	74.5±0.2	81.4±0.2	<.001
SBP (mmHg)	117.9±0.3	107.1±0.3	125.0±0.5	<.001
DBP (mmHg)	77.6±0.2	70.3±0.2	76.6±0.3	<.001
FPG (mmol/l)	5.18±0.01	4.97±0.01	5.24±0.01	<.001
Total cholesterol (mmol/l)	4.83±0.02	4.58±0.02	5.22±0.02	<.001
Triglyceride (mmol/l)*	1.40(1.37–1.43)	0.89(0.87–0.90)	1.29(1.26–1.33)	<.001
HDL cholesterol (mmol/l)	1.29±0.01	1.48±0.01	1.38±0.01	<.001
LDL cholesterol(mmol/l)	2.79±0.02	2.62±0.02	3.17±0.02	<.001
Current smoker (%)	47.7(0.9)	7.01(0.6)	5.5(0.7)	<.001
Heavy drinker (%)	17.7(0.6)	2.8(0.3)	1.0(0.3)	<.001
Regular exercise (yes,%)	27.4(0.8)	21.9(0.9)	23.0(1.2)	<.001
Fasting insulin (μIU/mL)*	10.3(10.1–10.4)	10.2(10.0–10.3)	10.3(10.1–10.5)	0.47
HOMA-IR*	2.11(2.07–2.15)	2.0(1.97–2.04)	2.14(2.2–2.19)	<.001
Metabolic syndrome (%)	20.6(0.7)	8.9(0.6)	40.4(1.2)	<.001
Abdominal obesity	23.0(0.8)	25.0(0.9)	56.3(1.3)	<.001
Triglyceride	36.2(0.9)	11.4(0.6)	36.4(1.2)	<.001
HDL cholesterol	20.6(0.7)	27.7(0.9)	46.7(1.1)	<.001
Blood pressure	35.2(0.9)	9.3(0.5)	53.4(1.3)	<.001
IFG	23.0(0.8)	10.0(0.6)	25.8(1.0)	<.001

Data are presented as the means ± standard error (SE) for continuous variables, or as proportions (SE) for categorical variables.

*geometric mean (95% confidence interval)

BMI: body mass index; WC: waist circumference; SBP: systolic blood pressure; DBP: diastolic blood pressure; FPG: fasting plasma glucose; HDL: high density lipoprotein; LDL: low density lipoprotein; IFG: impaired fasting glucose; HOMA-IR: homeostasis model assessment of insulin resistance.

## Results

The baseline clinical characteristics of the study population are shown in Table 1. Subjects were subdivided into men, premenopausal women, and postmenopausal women. Insulin resistance probably increases in women after menopause because of estrogen deficiency and increased visceral adipose tissue [6, 16]. Thus, subjects were divided into three groups for determining more accurate HOMA-IR cut-off values. The mean ages of men, premenopausal women, and postmenopausal women were 42.5±0.3, 35.2±0.2, and 61.7±0.3 years, respectively. WC, diastolic BP, and triglycerides were higher in men than in women. Systolic BP and fasting plasma glucose showed the highest levels in postmenopausal women. HDL-cholesterol was lowest in men. The mean HOMA-IR was 2.11 (2.07–2.15) for men, 2.0 (1.97–2.04) for premenopausal women, and 2.14 (2.2–2.19) for postmenopausal women.

The prevalence of metabolic syndrome was 20.6% in men, 8.9% in premenopausal women, and 40.4% in postmenopausal women (Table 1). We identified the proportion of the five components of metabolic syndrome according to each group. In men, high TG (36.2%) and BP (35.2%) accounted for a large percentage. Low HDL-cholesterol (27.7%) and abdominal obesity (25.0%) accounted for a large proportion in premenopausal women. Abdominal obesity (56.3%) and high BP (53.4%) accounted for a large proportion in postmenopausal women.

**Fig 1:**
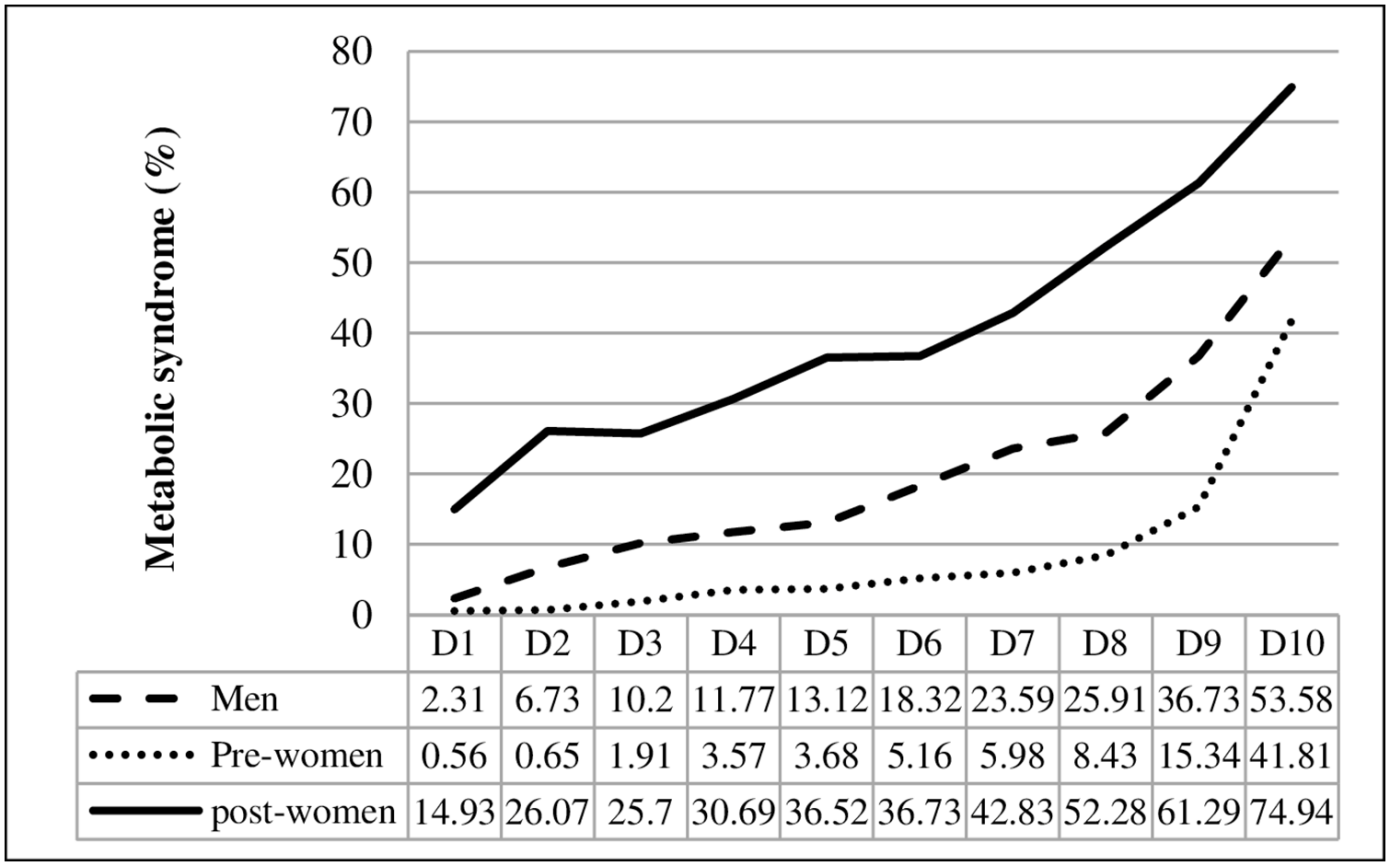
Prevalence of metabolic syndrome according to HOMA-IR. HOMA-IR: homeostasis model assessment of insulin resistance; Pre-women: premenopausal women; Post-women: postmenopausal women.

HOMA-IR values were divided into 10 deciles to identify the prevalence of metabolic syndrome according to HOMA-IR level (Table 2). Fig 1 shows the proportion of metabolic syndrome according to HOMA-IR. The prevalence of metabolic syndrome increased as HOMA-IR increased in all three groups. In men and premenopausal women, metabolic syndrome increased sharply at the ninth to tenth deciles of HOMA-IR (HOMA-IR 3.53 in men, 3.24 in premenopausal women). In postmenopausal women, metabolic syndrome increased continuously from the seventh decile of HOMA-IR (HOMA-IR 2.32–2.56).

**Table 2:** HOMA-IR values divided by 10 decile.

	D1	D2	D3	D4	D5	D6	D7	D8	D9	D10
Men	-1.24	1.24–1.47	1.47–1.68	1.68–1.85	1.85–2.04	2.04–2.27	2.27–2.53	2.53–2.89	2.89–3.53	3.53-
Pre-women	-1.24	1.24–1.45	1.45–1.62	1.62–1.77	1.77–1.94	1.94–2.12	2.12–2.35	2.35–2.66	2.66–3.24	3.24-
Post-women	-1.30	1.30–1.54	1.54–1.73	1.73–1.91	1.91–2.11	2.11–2.32	2.32–2.56	2.56–2.93	2.93–3.51	3.51-

HOMA-IR: homeostasis model assessment of insulin resistance; Pre-women: premenopausal women; Post-women: postmenopausal women

**Fig 2:**
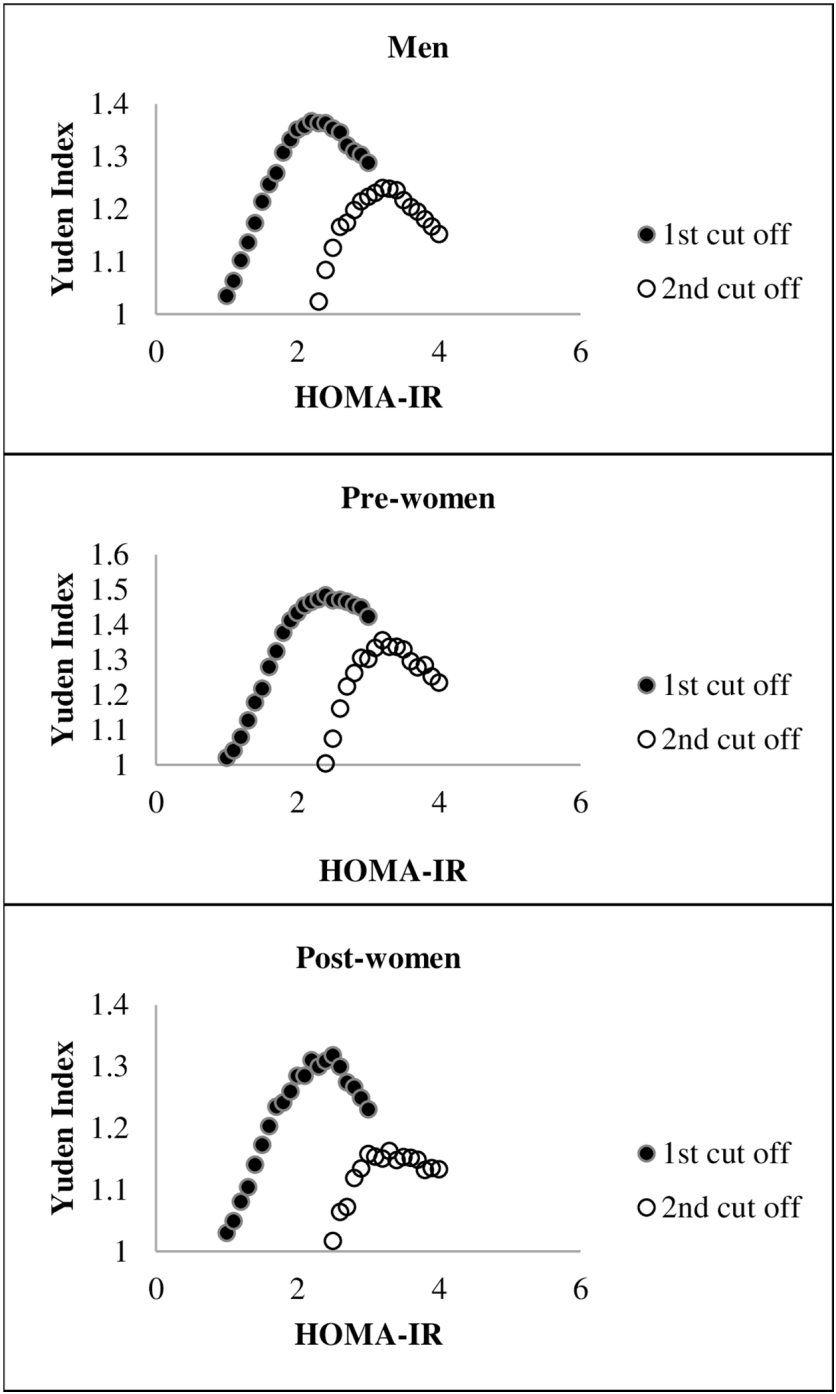
First and second cut-off values of HOMA-IR in men, premenopausal women and postmenopausal women. The Youden index, calculated as (sensitivity + specificity − 1) was estimated to determine optimal cut-off values. After those subjects with a HOMA-IR lower than the first HOMA-IR cut-off value had been excluded, the data were recalculated to obtain the second cut-off. The first cut-off values in men, premenopausal women, and postmenopausal women were 2.23 (AUC 0.75, sensitivity 70.6%, specificity 66.9%), 2.39 (AUC 0.82, sensitivity 72.3%, specificity 76.4%), and 2.48 (AUC 0.71, sensitivity 51.9%, specificity 80.2%). The second cut-off values in men, premenopausal women, and postmenopausal women were 3.23 (AUC 0.65, sensitivity 48.2%, specificity 76.5%), 3.20 (AUC 0.71, sensitivity 64.8%, specificity 70.8%), and 3.28 (AUC 0.61, sensitivity 45.8%, specificity 71.6%). HOMA-IR: homeostasis model assessment of insulin resistance; Pre-women: premenopausal women; Post-women: postmenopausal women.

ROC analysis was used to define HOMA-IR cut-off value for metabolic syndrome. The first cut-off values in men, premenopausal women, and postmenopausal women were 2.23 (sensitivity 70.6%, specificity 66.9%), 2.39 (sensitivity 72.3%, specificity 76.4%), and 2.48 (sensitivity 51.9%, specificity 80.2%), respectively (Fig 2). Based on the first HOMA-IR cut-off value, the prevalence of metabolic syndrome was 22.9% in men, 13.7% in premenopausal women, and 51.6% in postmenopausal women. The second cut-off values in men, premenopausal women, and postmenopausal women were 3.23 (sensitivity 48.2%, specificity 76.5%), 3.20 (sensitivity 64.8%, specificity 70.8%), and 3.28 (sensitivity 45.8%, specificity 71.6%). Based on the second HOMA-IR cut-off value, the prevalence of metabolic syndrome was 50.8% in men, 42.5% in premenopausal women, and 71.6% in postmenopausal women.

Table 3 shows the OR (95% CI) for each metabolic syndrome component according to HOMA-IR cut-off value after adjustment for age, BMI, and lifestyle factors (current smoking, heavy drinker, exercise). Results showed that increased fasting glucose had the highest OR value. The OR of metabolic syndrome according to HOMA-IR first cut-off value was 2.44 for men (95% CI = 1.98–3.00), 2.47 for premenopausal women (95% CI = 1.62–3.77), and 2.17 for postmenopausal women (95% CI = 1.65–2.85).

**Table 3:** Multivariate OR (95% CI) of metabolic syndrome component according to HOMA-IR cut off value.

	Men			Premenopausal women			Postmenopausal women		
	Ref	1^st^ cut off	2^nd^ cut off	Ref	1^st^ cut off	2^nd^ cut off	Ref	1^st^ cut off	2^nd^ cut off
AO	1	1.51(1.13–2.01)	2.32(1.70–3.16)	1	1.55(1.12–2.15)	1.59(1.05–2.40)	1	1.24(0.85–1.81)	2.41(1.34–4.33)
TG	1	2.02(1.68–2.42)	2.59(1.98–3.39)	1	2.18(1.61–2.96)	3.63(2.42–5.45)	1	1.86(1.45–2.38)	2.18(1.57–3.03)
HDL	1	1.48(1.20–1.82)	1.69(1.28–2.24)	1	1.08(0.85–1.38)	1.73(1.31–2.28)	1	1.36(1.07–1.73)	1.15(0.84–1.58)
BP	1	1.40(1.18–1.67)	1.57(1.21–2.03)	1	1.15(0.79–1.67)	1.97(1.33–2.93)	1	1.39(1.09–1.78)	2.14(1.43–3.20)
IFG	1	3.25(2.61–4.06)	7.53(5.76–9.84)	1	3.14(2.22–4.44)	11.1(7.64–16.0)	1	3.02(2.31–3.96)	8.58(6.18–11.9)
MS	1	2.45(1.99–3.02)	4.91(3.69–6.53)	1	2.47(1.62–3.77)	8.96(5.86–13.7)	1	2.17(1.65–2.85)	4.25(2.79–6.46)

All values were adjusted by age, Body mass index and lifestyle factors (current smoking, heavy drinker, regular exercise)

OR: odds ratio; CI: confidence interval; HOMA-IR: homeostasis model assessment of insulin resistance; AO: abdominal obesity; TG: triglyceride; HDL: high density lipoprotein; BP: blood pressure; IFG: impaired fasting glucose; MS: metabolic syndrome

## Discussion

HOMA-IR is a simple, less invasive, inexpensive and useful method to measure insulin resistance. Insulin resistance has a close association with cardiovascular risk factors and it is similar to the metabolic syndrome component [17, 18]. So, we calculated the HOMA-IR cut-off values for predicting metabolic syndrome in Koreans. In the present study, the first HOMA-IR cut-off values in men, premenopausal women, and postmenopausal women were 2.23, 2.39, and 2.48. The second cut-off value was around 3.2 in all three groups. Several other studies have attempted to define cut-off values of HOMA-IR using the ROC curves. The HOMA-IR cut-off values in Japanese, Iranian and Spanish subjects were 1.7–2.0 [6, 8, 19] These values were lower than first cut-off value of HOMA-IR in Korea. In Portuguese and Brazilian studies, the HOMA-IR cut-off values were 2.4–2.7, and it was similar to Korea [7, 20] However, the cut-off value in 1,854 Mexican Americans was 3.80 (specificity = 0.778, sensitivity = 0.616) [21]. The values showed variability by race and ethnicity. Thus, the ‘best’ cut-off for insulin resistance may need to be measured by race or country.

Additionally, the HOMA-IR cut-off value has shown different results depending on gender. In the present study, first cut-off value in women was little higher than in men. Furthermore, the cut-off values in the same age group were higher in women than in men. (Data not shown in tables.) Similar results were observed in Iran (1.7 in men and 1.8 in women), Spain (1.85 in men and 2.07 in women), and China (using 75^th^ percentile for threshold of HOMA-IR, 2.48 in men and 2.67 in women) [6, 10, 22]. However, some studies showed that there were no differences in values between genders [19, 23]. The different results of each study can occur due to definitions of the metabolic syndrome and characteristics of subjects.

In the present study, the mean HOMA-IR values in men decreased with age, the mean HOMA-IR values in women showed a tendency to increase from 50 years of age. (Data not shown in tables.) Also, previous other studies showed that the HOMA-IR value increases significantly from 50 years of age in non-diabetic women [6, 10, 24]. Thus, in this study, we analyzed separately for premenopausal and postmenopausal women. Causes of the deterioration of insulin resistance in women more than 50 years old may involve estrogen deficiency. Physiological levels of estradiol plays a role in maintaining insulin sensitivity [25, 26]. Estrogen deficiency due to menopause affects glucose and insulin metabolism [16, 27] and body fat distribution [28]. Thus, estrogen deficiency and central obesity in postmenopausal women may aggravate insulin resistance and metabolic syndrome.

Some studies have reported HOMA-IR cut-off values in non-diabetic healthy Korean people [11, 12]. The results showed that the cut-off value of HOMA-IR for metabolic syndrome by the ATP III criteria was 2.3–2.5. It was similar to our results. However, to our knowledge, no reported study has identified second cut-off values of HOMA-IR or performed subgroup analysis by gender and menopausal status in Koreans before. In the present study, there were great differences in the prevalence of metabolic syndrome according to HOMA-IR in the three groups. The prevalence of metabolic syndrome at the second cut-off value of HOMA-IR was 40–50% in men and premenopausal women. However, in postmenopausal women, the prevalence of metabolic syndrome at the first cut-off value was 51.6%. Considering the prevalence of metabolic syndrome, second cut-off value (about 3.2) is more clinically useful in men and premenopausal women and first cut-off value (about 2.5) is useful in postmenopausal women. Thus, we have a choice between the first and second cut-off values of HOMA-IR in consideration of the gender, menopausal status, and the prevalence of metabolic syndrome.

Previous studies about HOMA-IR in non-diabetic Koreans showed that the prevalence of metabolic syndrome was 10–15% [11, 23]. However, the prevalence of metabolic syndrome in our study was 21.5% (men 20.6%, women 22.1%). The first possible cause of this difference is the different diagnostic criteria for metabolic syndrome. Therefore, there may have been an increase in the prevalence of metabolic syndrome by the reduced fasting glucose standard (6.1 → 5.6 mmol/L). Additionally, the other studies were single center studies and subjects who visited for medical check-ups were assessed. Thus, relatively healthy people may have been included in the existing research. Also, the number of people with metabolic syndrome in Korea is increasing, due to lifestyle changes [29].

There are several strengths of the present study. First, this study used a large, nationally representative, population-based data set in Korea. Second, subjects were divided into three groups by gender and menopausal status. We could identify the HOMA-IR cut-off value and prevalence of metabolic syndrome for each group. Third, both the first and second HOMA-IR cut-off values were calculated. Thus, the values can be used differently, depending on the clinical situation. However, the study also has some limitations. First, this study was cross-sectional in design. Thus, insulin resistance was identified by HOMA-IR based on single tests of fasting blood glucose and insulin. Another limitation is that the subjects consisted of only non-diabetic patients. Insulin resistance in diabetic patients may vary depending on the duration of diabetes mellitus, use of oral hypoglycemic agents or insulin, and glycemic control status. However, these are limitations due to the nature of the data set.

## Conclusion

In conclusion, the first cut-off values of HOMA-IR were 2.2–2.5 and the second cut-off value of HOMA-IR was 3.2 in Koreans. The distribution of HOMA-IR and prevalence of metabolic syndrome showed differences according to gender and menopausal status. When we apply HOMA-IR, we should consider gender, menopausal status, and the prevalence of metabolic syndrome.
